# Characterization of Early Life Stress-Affected Gut Microbiota

**DOI:** 10.3390/brainsci11070913

**Published:** 2021-07-10

**Authors:** Noriyoshi Usui, Hideo Matsuzaki, Shoichi Shimada

**Affiliations:** 1 Department of Neuroscience and Cell Biology, Graduate School of Medicine, Osaka University, Osaka 565-0871, Japan; shimada@anat1.med.osaka-u.ac.jp; 2 United Graduate School of Child Development, Osaka University, Osaka 565-0871, Japan; matsuzah@u-fukui.ac.jp; 3 Global Center for Medical Engineering and Informatics, Osaka University, Osaka 565-0871, Japan; 4 Addiction Research Unit, Osaka Psychiatric Research Center, Osaka Psychiatric Medical Center, Osaka 541-8567, Japan; 5 Division of Development of Mental Functions, Research Center for Child Mental Development, University of Fukui, Fukui 910-1193, Japan; 6 Life Science Innovation Center, University of Fukui, Fukui 910-1193, Japan

**Keywords:** early life stress, social isolation, social behavior, gut microbiota, *Bacteroidales*, *Clostridiales*

## Abstract

Early life stress (ELS), such as neglect and maltreatment, exhibits a strong impact on the mental and brain development of children. However, it is not fully understood how ELS affects the body and behavior of children. Therefore, in this study, we performed social isolation on weaned pre-adolescent mice and investigated how ELS could affect gut microbiota and mouse behavior. Using the metagenomics approach, we detected an overall ELS-related change in the gut microbiota and identified *Bacteroidales* and *Clostridiales* as significantly altered bacterial groups. These metagenomic alterations impaired social behavior in ELS mice, which also correlated with the abundance of *Bacteroidales* and *Clostridiales*. Our results demonstrate that ELS alters the gut microbiota and reduces social behavior in adolescent mice.

## 1. Introduction

The environment is a crucial factor for providing optimal growth and health conditions for children, including social, cognitive, or immune-system-related aspects [1,2,3,4]. In terms of brain development, the childhood environment impacts brain architecture, synaptic plasticity, and mental development [5,6]. Early life stress (ELS) suffered during childhood, such as neglect and maltreatment, has a critical influence on the mental and brain development of children [7,8]. For example, maltreatment is one risk factor for post-traumatic stress disorder (PTSD) and results in reduced volumes of the anterior cingulate gyrus, the ventromedial and dorsomedial cortex, and the hippocampus [7,8].

Previous studies in mice reported that mouse pups socially isolated during the pre-adolescent to adolescent period (postnatal days (P) 21–35) displayed altered social behavior and reduced myelination in the brain [9]. The effect of ELS on the growth of children represents a serious problem, as children need to receive necessary healthy inputs during this critical developmental period. Those who do not receive optimal inputs during this period would be affected in the long term.

Recent studies have reported that the gut–brain axis affects brain development and its function [10]. Gut microbiota is important for the growth and functioning of the brain and body through metabolism. We have previously reported that changes in the gut microbiota of pregnant mice result in changes in the gut microbiota of the offspring and increases of anxious behavior [11]. Alterations of gut microbiota in several mental disorders, such as autism spectrum disorder (ASD), schizophrenia, and depression, have also been reported [12,13,14]. In particular, patients with ASD and schizophrenia show impairment of social behaviors as a symptom. The alterations of many bacterial abundances such as *Bifidobacterium, Lactobacillus, Bacteroides, Ruminococcaceae, or Verrucomicrobia* have been reported in patients with ASD or schizophrenia [12,14,15,16], which suggests the possibility that the gut–brain axis may affect social behavior. In addition, stress reportedly affects gut microbiota [17]. Therefore, we hypothesized that ELS affects gut microbiota, which results in changes in individual behavior via the gut–brain axis. To address this question, we aimed to investigate how ELS could affect the gut microbiota and behaviors of the mice, especially social behavior and cognition during adolescence by using the mouse as a model.

In this study, we performed social isolation on weaned pre-adolescent mice to analyze the effect of ELS on gut microbiota. Using the feces of these mice, we conducted 16S ribosomal RNA sequencing (16S rRNA sequencing) to characterize their gut microbiota. We also investigated whether the alterations in the gut microbiota correlate with murine social behavior.

## 2. Materials and Methods

### 2.1. Mice

Wild-type C57BL/6N (Japan SLC Inc., Shizuoka, Japan) mice were used. The 25 mice used in this study were housed in the barrier facilities of University of Fukui under 50% humidity and a 12:12 h light:dark cycle (8:00 to 20:00) and given free access to water and food. Social-isolation-induced ELS was performed as described previously [9], with modifications. Briefly, experimental and control mice were used from the litters of the same mother. For ELS (via social isolation), 5 male mice were individually housed using a filter cap (#CL-4150, CLEA Japan, Inc., Tokyo, Japan) immediately after the weaning at P21 until P53 (Figure S1). For controls, 4 male mice were co-housed in the same cage without a filter cap, and 5 randomly selected mice (1 mouse/cage) were used for the experiments (Figure S1). All procedures were performed according to the ARRIVE guidelines and relevant official guidelines under the approval (#27-010) of the Animal Research Committee of the University of Fukui.

### 2.2. 16S Ribosomal RNA Sequencing (16S rRNA-seq)

Fresh fecal samples were collected at P53 before behavioral test at morning time (9:00 to 10:00). Feces from a single cage were counted as one biological sample; thus, 5 cages/conditions were used. The 16S rRNA-seq was performed as a service provided by the APRO Life Science Institute (Tokushima, Japan). Genomic DNA was extracted from mice fecal samples using the Quick-DNA Fecal/Soil Microbe Miniprep Kit (# D6010, ZYMO research, Irvine, CA, USA) according to the manufacturer’s instructions. The V3-V4 regions of the 16S rDNA were PCR-amplified using specific primers and purified using AMPure XP beads (#A63882, Beckman Coulter, Brea, CA, USA). The amplicons were PCR-indexed and subjected to AMPure XP bead purification. The cDNA library quality was evaluated by 2100 Bioanalyzer using the Agilent High Sensitivity DNA Kit (#5067-4626, Agilent, Santa Clara, CA, USA). Libraries were sequenced as 301 bp paired-end on Illumina MiSeq.

### 2.3. 16S rRNA-seq Data Analysis

Paired-end reads were quality-filtered and assembled using FLASH [18]. Demultiplexed sequences were denoised with DADA2 [19]. Then, the sequences were assigned using Greengenes 13_8 OTUs full-length sequences for operational taxonomic unit (OTU) clustering and taxonomy analyses. The metagenomic analyses for visualizing bacterial taxa and analyzing principal coordinates analysis (PCoA) were conducted using QIIME 2 [20]. To identify bacterial taxa with differential representation between groups at genus or higher levels, we conducted edgeR (false discovery rate (FDR) <0.05 cutoff) [21] and linear discriminant analysis effect size (LEfSe) [22].

### 2.4. Three-Chamber Social Interaction Test

Sociability and social novelty behaviors were assessed using a three-chamber box (W610 × D400 × H220 mm) with an infrared video camera system (O’Hara & Co., Ltd., Tokyo, Japan) at P53 after collecting fecal samples. For the first trial, empty wired cages were placed into both chambers for habituation. For the second trial, a stranger male mouse (mouse A) was placed into a wired cage of the right chamber to examine sociability. For the third trial, mouse A was kept in the same wired cage as a familiar mouse, and a novel stranger male mouse (mouse B) was placed into a wired cage of the left chamber to examine social novelty. Each trial was examined for 5 min after which the interactions with the targets were scored using an infrared video camera system (O’Hara & Co., Ltd., Tokyo, Japan). An experimenter blind to genotypes performed all behavioral tests. All behavioral tests were performed between 10:00 and 16:00 h.

### 2.5. Statistical Analysis

All behavioral data are represented as the means of biologically independent experiments with ± standard errors of the mean (SEM). The statistical analyses (independent samples *t*-test and Pearson’s r) were performed using the GraphPad Prism 9 software. Asterisks indicate *p*-values (** *p* < 0.001, ** *p* < 0.01, * *p* < 0.05) with *p* < 0.05 being considered as the threshold of statistical significance.

## 3. Results

### 3.1. Effects of ELS on Gut Microbiota

**Figure 1:**
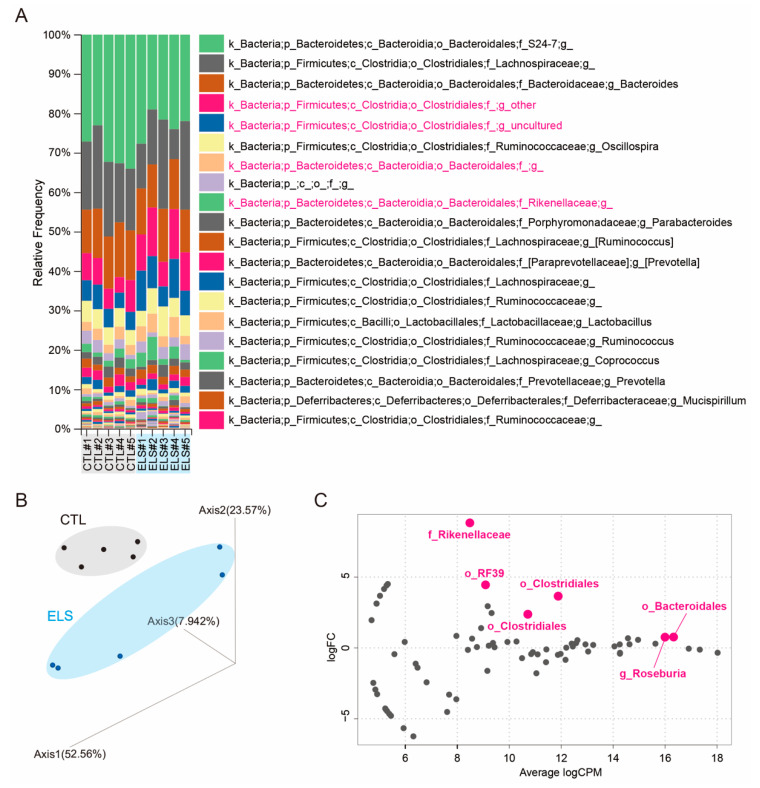
Early life stress-influenced gut microbiota alterations in mice. (**A**) The top 20 relative abundant taxa were shown at the genus level. (**B**) Principal coordinates analysis (PCoA) on the OTU level. (**C**) MA plot showing significant difference in the gut microbiota in early life stressed (ELS) mice. The significantly altered gut bacteria are shown in magenta. CTL, control mice; ELS, early life stressed mice, *n* = 5/condition.

To investigate the impact of ELS on gut microbiota and behaviors in adolescent mice at early stages, we performed social isolation immediately after the weaning on P21 and maintained it until P53 (Figure S1). After the social isolation periods, we first performed 16S rRNA-seq to identify a potential ELS-induced alteration of the gut microbiota. We targeted the V3–V4 regions of the 16S rRNA to identify individual microorganisms for metagenomic analysis and detected the alteration of the gut microbiota. After the OTU analysis, the abundant taxa were identified at the genus level in individual control and ELS mice using QIIME 2 (Figure 1A and Figure S2). PCoA of metagenome indicated the separation between the control and ELS clusters (Figure 1B). To identify the alteration of gut microbiota, we analyzed the statistically significant differences in the gut microbiota at the genus level between the groups using edgeR. Then, we identified six gut bacteria as differential gut microbiota (Figure 1C). *Bacteroidales* (log fold change (logFC) = 3.65, FDR = 0.0011), *Roseburia* (logFC = 8.81, FDR = 0.0608), *Clostridiales* (logFC = 0.77, FDR = 0.1076; logFC = 0.76, FDR = 0.1373), *Mollicutes RF39* (logFC = 4.45, FDR = 0.4075), and *Rikenellaceae* (logFC = 2.37, FDR = 0.4283) were identified as gut bacteria affected by ELS (Figure 1C).

### 3.2. LEfSe in Altered Gut Microbiota by ELS

**Figure 2:**
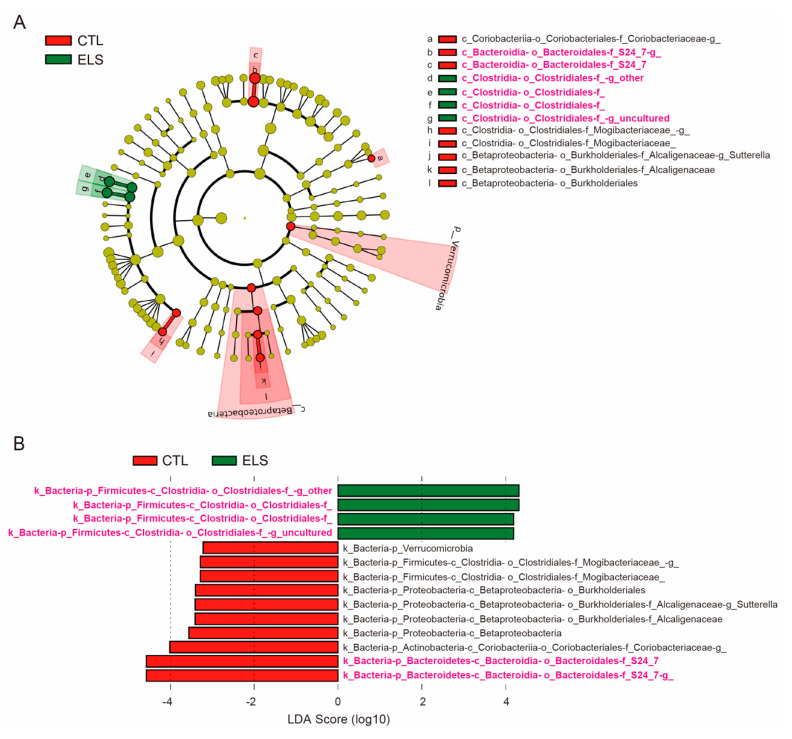
Linear discriminant analysis effect size in gut microbiota. Linear discriminant analysis effect size (LEfSe) showing relative taxonomic abundance at the genus level. (**A**) Cladogram by LEfSe. (**B**) LDA scores for differential gut microbiota by LEfSe. The *s*ignificantly altered gut bacteria in 16S rRNA-seq are shown in magenta. CTL, control mice; ELS, early life stressed mice, *n* = 5/condition.

We further analyzed gut microbiota using LEfSe to identify taxa when the microbial distribution was significantly and statistically different between groups with the defined microbial distribution. By LEfSe, 14 gut bacteria were identified at the genus level for relative taxonomic abundance (Figure 2A,B). Linear discriminant analysis (LDA) scores for differential gut microbiota uncovered that 10 gut bacteria (*Bacteroidales S24-7* (LDA scores = 4.5624, 4.585), *Coriobacteriaceae* (LDA score = 4.0136), *Betaproteobacteria* (LDA score = 3.5646), *Alcaligenaceae* (LDA score = 3.4059), *Sutterella* (LDA score = 3.415), *Burkholderiales* (LDA score = 3.4014), *Mogibacteriaceae* (LDA scores = 3.2971, 3.2834), and *Verrucomicrobia* (LDA score = 3.22)) were significantly reduced in the ELS mice (Figure 2B and Figure S3). In contrast, 4 gut bacteria (*Clostridiales* (LDA scores = 4.3311, 4.3084, 4.1905, 4.1995)) were significantly increased in the ELS mice (Figure 2B and Figure S3). Among those 14 gut bacteria identified by LEfSe, 5 differential gut bacteria (*Bacteroidales* and *Clostridiales*) were identified using edgeR, which were only observed in the gut bacteria increased by ELS (Figure 1 and Figure 2).

### 3.3. Decreased Social Behavior Due to ELS

**Figure 3:**
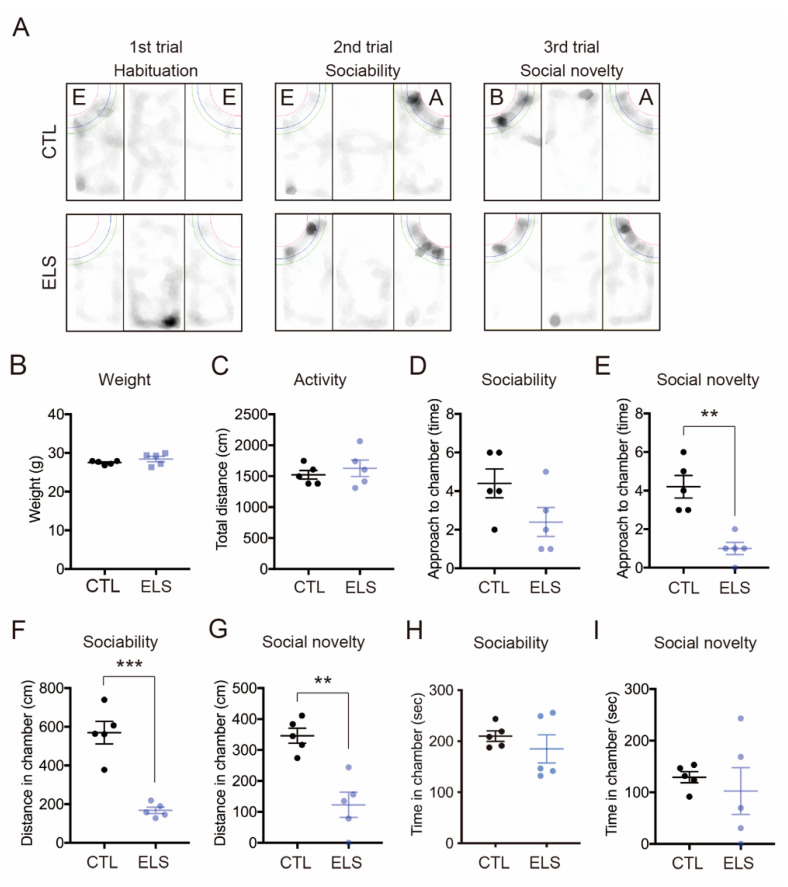
ELS distinctively affected social behaviors in mice. (**A**) Mount graph showing behavioral trace examples in the three-chamber social interaction test. E, empty; A: stranger mouse 1; B: stranger mouse 2. (**B**–**I**) Reduced social behavior was observed in ELS mice. There was no difference in weight (**B**) or total distance (**C**) in the three-chamber social interaction test. (**D**–**I**) The number of approaches (**D**), time (**F**), and distance (**H**) is shown during the 2nd trial (sociability behavior) (**D**,**F**,**H**) and the 3rd trial (social novelty behavior) (**E**,**G**,**I**), respectively. CTL, control mice; ELS, early life stressed mice. Data are represented as the means (±SEM). Asterisks indicate *** *p* < 0.001, ** *p* < 0.01, unpaired *t*-test, *n* = 5/condition.

After the social isolation periods, we examined the social behaviors during the three-chamber social interaction test (Figure 3A). Social isolation did not alter the mouse weight (CTL = 27.5 ± 0.22, ELS = 28.42 ± 0.70, 95% confidence interval (Cl) = −0.7783 to 2.618, *p* = 0.25) and locomotion activity (CTL = 1522 ± 70.12, ELS = 1628 ± 132.70, 95% Cl = −240.5 to 451.5, *p* = 0.50) (Figure 3B,C). Compared with control mice, ELS mice showed significant reductions in sociability and social novelty behaviors (Figure 3D–I). In the sociability period, we observed significantly reduced measurements of distance (CTL = 570 ± 58.03, ELS = 168 ± 16.27, 95%Cl = −540.9 to −263.0, *p* = 0.0002) around the stranger mouse in the ELS mice (Figure 3F), but not in approach (CTL = 4.4 ± 0.75, ELS = 2.4 ± 0.75, 95%Cl = −4.440 to 0.4404, *p* = 0.09) or time (CTL = 209.9 ± 10.18, ELS = 184.9 ± 27.61, 95%Cl = −92.87 to 42.87, *p* = 0.42) (Figure 3D,H). In the social novelty period, reduced measurements of approach (CTL = 4.2 ± 0.58, ELS = 1 ± 0.32, 95%Cl = −4.730 to −1.670, *p* = 0.0013) and distance (CTL = 345.9 ± 24.21, ELS = 122.8 ± 40.45, 95%Cl = −332.0 to −114.1, *p* = 0.0015) to a stranger mouse were also observed in the ELS mice compared with the control (Figure 3E,G), but not time (CTL = 129.2 ± 10.78, ELS = 102.3 ± 45.21, 95%Cl = −134.1 to 80.27, *p* = 0.58) (Figure 3I). These results indicate that ELS in pre-adolescent mice impacts social behaviors via social isolation.

### 3.4. Correlation between Altered Gut Bacteria and Social Behavior

**Figure 4:**
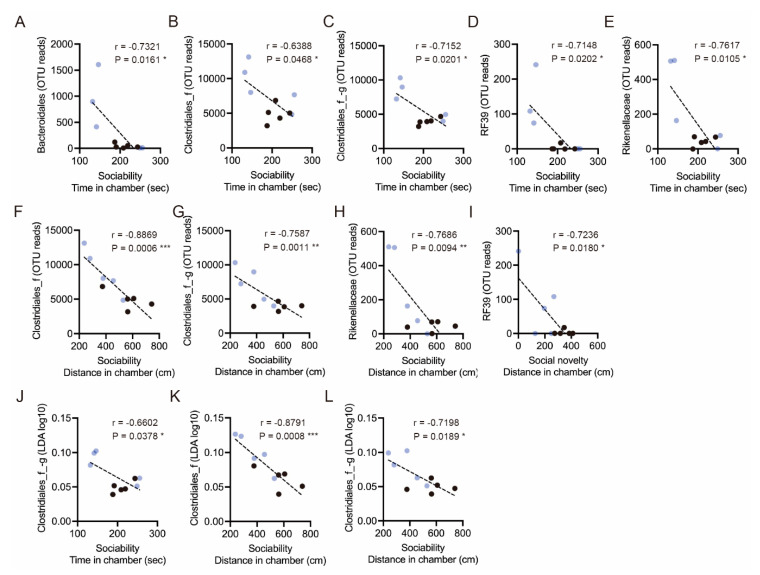
Correlations between gut microbiota and social behaviors. (**A**–**E**) Correlations between the OTU reads of *Bacteroidales*, *Clostridiales, Mollicutes RF39,* and *Bacteroidales Rikenellaceae* with time in the chamber during the sociability period. (**F**–**H**) Correlations between the OTU reads of *Clostridiales* and *Bacteroidales Rikenellaceae* with distance in the chamber. (**I**) A correlation between the OTU reads of *Mollicutes RF39* with distance in the chamber during the social novelty period. (**J**) A correlation between the LDA score of *Clostridiales* with time in chamber during the sociability period. (**K**,**L**) Correlations between the LDA scores of *Clostridiales* with distance in the chamber. Individual black and blue dots indicate control and ELS mice, respectively. Asterisks indicate *** *p* < 0.001, ** *p* < 0.01, * *p* < 0.05, Pearson’s r, *n* = 10 (*n* = 5/condition).

Finally, we analyzed whether the ELS-altered gut microbiota correlates with social behavior. In the altered gut microbiota, we found that the OTU reads of *Bacteroidales* (r = −0.7321, *p* = 0.0161), *Clostridiales* (r = −0.6388, *p* = 0.0468; r = −0.7152, *p* = 0.0201)*, Mollicutes RF39* (r = −0.7148, *p* = 0.0202)*,* and *Bacteroidales Rikenellaceae* (r = −0.7617, *p* = 0.0105) correlated with social interaction time (Figure 4A–E). We also observed that the OTU reads of *Clostridiales* (r = −0.8869, *p* = 0.0006; r = −0.7587, *p* = 0.0011)*, Bacteroidales Rikenellaceae* (r = −0.7686, *p* = 0.0094)*,* and *Mollicutes RF39* (r = −0.7236, *p* = 0.0180) correlated with social interaction distance (Figure 4F–I). In addition, the LDA scores of *Clostridiales* (r = −0.6602, *p* = 0.0378; r = −0.8791, *p* = 0.0008; r = −0.7198, *p* = 0.0189) also correlated with time in social interaction time or distance (Figure 4J–L). These results indicate that bacteria, such as *Bacteroidales* and *Clostridiales,* are associated with murine social behaviors.

The above taken together, our study shows that social-isolation-related ELS in pre-adolescent mice impacts gut microbiota distribution and murine social behavior. Our characterization of the gut microbiota demonstrates that *Bacteroidales* and *Clostridiales,* identified by two different analyses, are among the ELS-affected bacterial groups of mouse gut microbiota.

## 4. Discussion

In this study, we show that ELS affects gut microbiota and social behaviors in mice. We found that a deficiency of forced social interactions after weaning significantly alters the distribution of gut microbiota in mice. Using two different analytical methods to explore the ELS-affected gut microbiota, we identified *Bacteroidales* and *Clostridiales* as commonly altered bacteria in ELS mice. We also observed impaired social behavior in ELS mice. The abundance of *Bacteroidales* and *Clostridiales* correlated with murine social behaviors, which indicates that ELS affects both gut microbiota and social behaviors in adolescent mice.

Stress is a well-known influencing factor of the microbiota–gut–brain axis that could potentially alter brain function and behavior [17]. It has been reported that *Bacteroidales* and *Clostridiales* were significantly reduced in social-defeat-stressed mice compared with controls [23]. In maternal separation at early postnatal periods (3 h per day from P2 to P14), a reduced abundance of *Bacteroidales S24–7* and an increased abundance of *Clostridiales vadinBB660* have been reported in postnatal rats [24]. Notably, the results related to *Bacteroidales S24–7* are consistent with our results (Figure 2), suggesting that it is indeed a bacterial group affected by ELS. In a similar model of pre-adolescent social isolation (P24–87) in lister hooded rats, hyperactivity and increased defecation frequency had been reported in open field trials [25]. In these rats, a reduction of *Clostridiales* abundance had been reported to be positively correlated with defecation frequency [25]. These findings, including ours, suggest that *Bacteroidales* and *Clostridiales* are stress-responsive bacteria that could potentially affect host behaviors.

Interestingly, the changes in the gut microbiota in this study closely resemble the reports of gut microbiota in ASD-affected subjects. ASD is a heterogeneous neurodevelopmental disorder that causes pervasive abnormalities in social communication as well as repetitive behaviors and restricted interests. The phenotype of impaired social behavior in ELS mice is similar only in terms of impaired social communication in ASD; therefore, the alterations of *Bacteroidales* and *Clostridiales* in ASD support our results. In fact, gastrointestinal disturbances are commonly reported in children with ASD, and reductions in the *Bacteroidetes* relative abundance and gastrointestinal disturbances in children with ASD have also been reported [26,27]. The abundance of *Bacteroides* and *Clostridiales* was significantly reduced in the ASD-like model valproic acid (VPA) treated male offspring [28]. In addition, a significant reduction of *Bacteroides fragilis* abundance was identified in the maternal immune activation (MIA) of ASD-like mouse model offspring [29]. Next, *Bacteroides fragilis* supplementation to MIA offspring rescued ASD-like behavioral abnormalities, such as ultrasonic vocal communication, repetitive behaviors, anxiety, and pre-pulse inhibition [29]. However, other studies reported opposite results, showing a high abundance of *Bacteroidetes* in children with ASD [30,31] or pervasive developmental disorder not otherwise specified (PDD-NOS) [30]. Moreover, the *Bacteroides* abundance significantly increased in ASD-like model BTBR mice compared with C57BL/6J mice [32]. Taken together, these studies demonstrate that *Bacteroidales* and *Clostridiales* are associated with ASD pathophysiology, suggesting that *Bacteroidales* and *Clostridiales*, which we identified, are involved in social behaviors.

Lastly, we acknowledge that our study has limitations in the identification of species due to the sample size and sequencing depth for the metagenomic analysis. Future studies should also consider the differences in the effects of ELS on mouse strain, gender, and various behaviors. The blood concentrations of cortisol and inflammatory cytokine should also be measured in order to quantify the degree of stress and inflammation in consideration of individual differences. Furthermore, uncovering of the role of *Bacteroidales* and *Clostridiales* in social behavior and gut microbiota should give rise to novel insights for understanding the gut–brain axis. Similarly, the analysis of blood metabolites will contribute to an understanding of the gut–brain axis.

## 5. Conclusions

In conclusion, our results demonstrate that ELS affects gut microbiota, such as *Bacteroidales* and *Clostridiales,* and mouse social behaviors, providing new insights into the relationship between the effects of ELS, gut microbiota, and potentially related behaviors.

## Data Availability

The data presented in this study are available on request from the corresponding author.

## Supplementary Materials

The following are available online at https://www.mdpi.com/article/10.3390/brainsci11070913/s1, Figure S1: Experimental design of this study, Figure S2: Early life stress induced alteration of gut microbiota composition, Figure S3: Relative taxonomic features of gut microbiota in ELS mice.
