# Supplementary material for: Characterization of Early Life Stress-Affected Gut Microbiota
Source: Brain Sci. 2021 Jul 10;11(7):913. doi: 10.3390/brainsci11070913 (PMC8306161; doi:10.3390/brainsci11070913)
Supplement: Supplementary file 1 [file brainsci-11-00913-s001.zip › brainsci-1279375-supplementary.pdf]

brain sciences

MDPI

Supplementary Materials

Characterization of early life stress-affected gut microbiota

Noriyoshi Usui, Hideo Matsuzaki and Shoichi Shimada

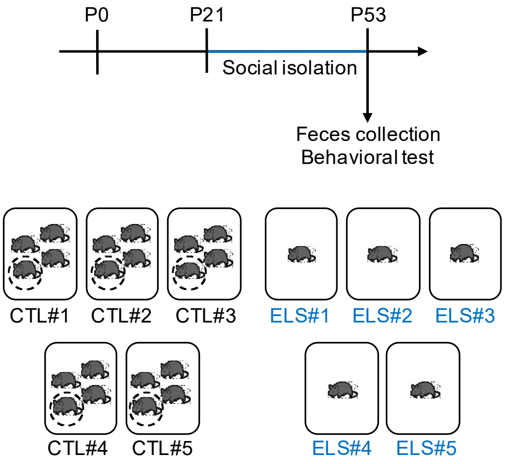

Figure S1. Schematic diagram of experimental design. For ELS mice, male mice were individually housed immediately after the weaning at postnatal days 21 until P53. For control mice, 4 male mice were co-housed in the same cage after the weaning at P21. One mouse from a cage was randomly selected for behavioral test. In total, 25 male mice were used for this study. Fecal samples were collected at P53 before three-chamber social interaction test. Feces from a one cage were counted as a one biological sample, thus 5 cages/condition were used. *CTL, control mice; ELS, early life stressed mice.*

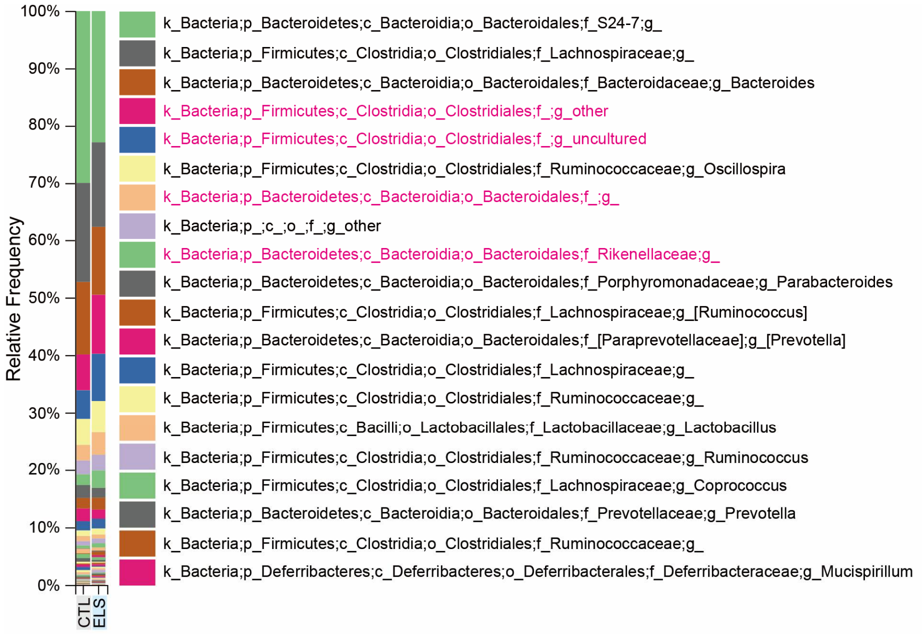

Figure S2. Early life stress induced alteration of gut microbiota composition. The top 20 relative abundant taxa were shown at genus level in intergroup comparative analysis. *CTL, control mice; ELS, early life stressed mice*, n =5/condition.

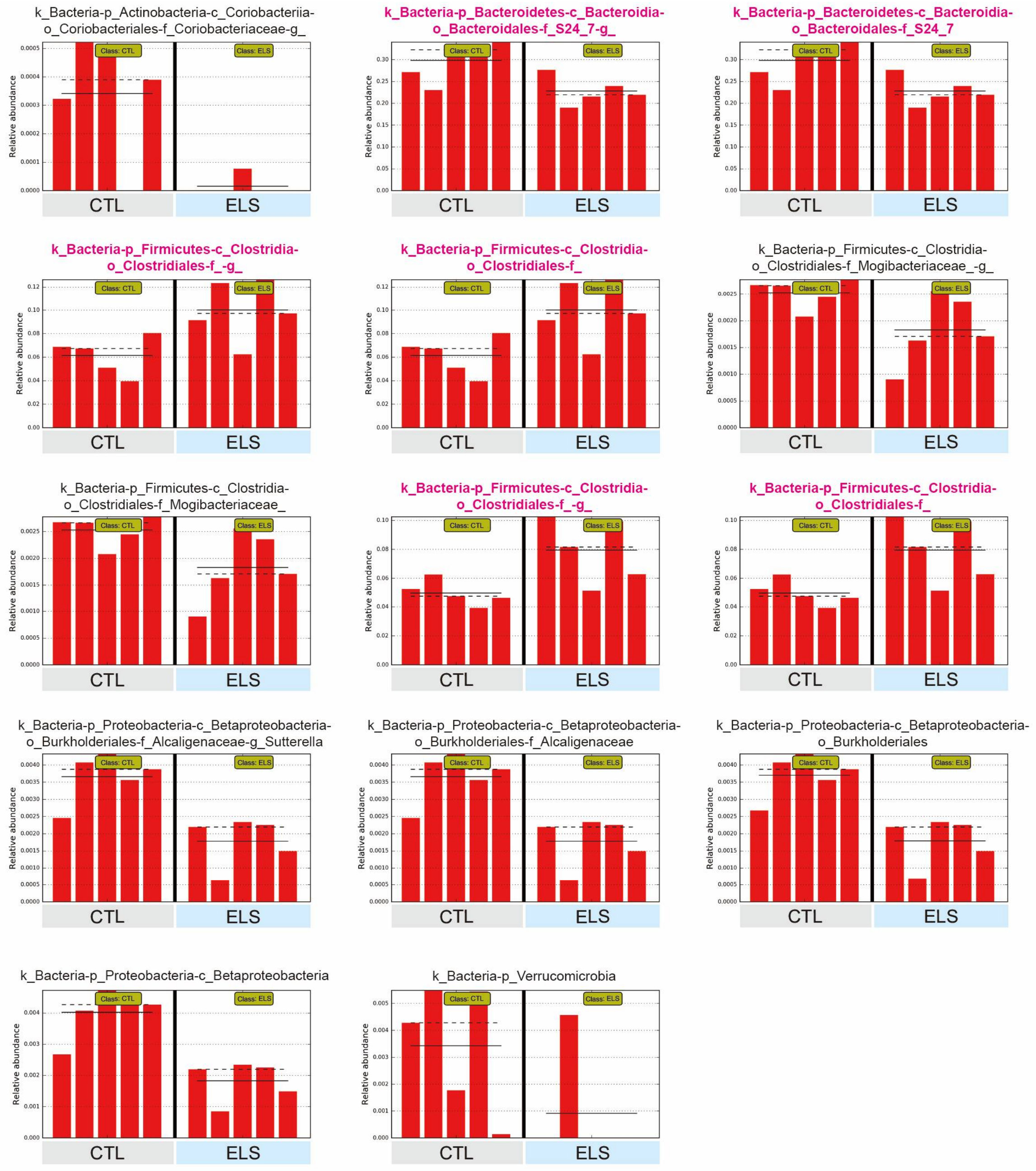

Figure S3. Each histogram showing relative taxonomic abundances at genus level by LEfSe (see also Figure 2). Significant altered gut bacteria in 16S rRNA-seq were shown in magenta. *CTL, control mice; ELS, early life stressed mice*, n=5/condition.
